# Student’s *t*-Distributed Extended Kalman Filter with Switch Factor for UWB Localization Under Colored Measurement Noise

**DOI:** 10.3390/mi16111231

**Published:** 2025-10-29

**Authors:** Yuan Xu, Haoran Yin, Maosheng Yang, Lei Deng, Mingxu Sun

**Affiliations:** 1 School of Electrical Engineering, University of Jinan, Jinan 250022, China; 2 School of Intelligent Equipment, Shandong University of Science and Technology, Taian 253034, China; 3 School of Electrical Engineering, Huayu University of Technology, Dezhou 253034, China

**Keywords:** Kalman filter, Student’s t distribution, UWB, robotic dog

## Abstract

To increase information accuracy when using ultrawide-band (UWB) localization for robotic dogs, we introduce a switching method for a Student’s *t*-distributed extended Kalman filter (EKF) that achieves UWB localization under colored measurement noise (CMN). First, a distributed UWB localization framework under CMN is designed, which can reduce the impact of CMN caused by carrier jitter on positioning accuracy. Then, a Student’s *t*-distributed EKF under CMN with a switch factor is proposed, which effectively improves the adaptability of the algorithm through adaptive selection of colored factors. Finally, experimental validation demonstrates the efficacy and high performance of the proposed method for two practical scenarios.

## 1. Introduction

In recent years, the proliferation of automation and intelligent control technologies has catalyzed the widespread adoption of mobile robotic systems across diverse applications, such as inspection [1,2,3] and agricultural [4,5] robotic systems, progressively displacing traditional human-operated processes. As accurate navigation and localization are fundamental requirements for autonomous mobile robot systems executing precision tasks, they constitute a prominent research focus in robotics [6,7]. As the application scope of quadruped mobile robots expands, the range of hazardous environments where they can replace human operations is gradually increasing, and the navigation environments are becoming increasingly complex [8]. For example, the emergence of urban canyons poses a challenge to the signal acquisition of pose measurement sensors [9]; the wall material of indoor navigation environments will affect the accuracy of positioning [10]; and the shaking of small carriers during operation has an impact on sensor measurement [11]. Consequently, existing navigation technologies and methods face several challenges [12]. Thus, the comprehensive study and systematic development of autonomous navigation algorithms and precise localization methods hold great research and practical importance for advancing mobile robot systems operating in complex, dynamic, and challenging operational environments characterized by uncertain conditions, sensor limitations, and environmental constraints. Such advances are valuable across diverse industrial sectors, service domains, and critical mission scenarios requiring reliable autonomous navigation capabilities.

Current research on mobile robot navigation and localization has been directed to multiple aspects, such as navigation and localization technologies and data fusion algorithms [13]. For the comprehensive development and systematic advancement of localization and positioning methods, numerous research attempts, diverse technical approaches, innovative algorithmic developments, and varied experimental investigations have been presented across different academic institutions, industrial research laboratories, and engineering development centers worldwide. For example, in [14], a pure pursuit path tracking algorithm optimized using the nondominated sorting genetic algorithm II for mobile robots is proposed. In this method, the accuracy of path tracking is improved by considering the dynamic characteristics and real-world operating conditions of mobile robots. The status and future development of BeiDou navigation satellite system (BDS) high-precision services are reviewed in [15]. A BDS–vision fusion method for precise pallet positioning and orientation estimation is proposed in [16], in which coordinate transformation algorithms enable the integration of BDS localization data to determine target pallet locations within the forklift navigation reference frame. Although the BDS allows us to provide stable position information in outdoor environments, its accuracy may reduce greatly when the BeiDou satellite signals are subjected to interference owing to phenomena such as the urban canyon effect. To prevent this problem, various short-distance localization technologies have been proposed. For example, an ultrahigh-frequency radio frequency identification (RFID) sensing system utilizing a coupled ring resonator for enhanced antenna functionality is proposed in [17]. In [18], a passive RFID-based indoor robot localization system using three known-position tags is proposed. A wireless-network-based approach for cooperative localization and environmental mapping of multirobot heterogeneous systems in uncharted operational domains formulated as a categorization task is proposed in [19]. An enhanced WiFi localization system is introduced to mitigate small-scale variability in wireless sensor measurements using soft computing [20]. However, RFID and WiFi technologies provide decimeter-level accuracy, being unable to meet the high-precision navigation and localization requirements of existing robots. To enhance the localization accuracy, the ultrawide band (UWB) has been considered [21], enabling affordable and precise localization for environments without coverage from a Global Navigation Satellite System. For instance, in [22], a multirobot cooperative localization framework for identifying UWB ranging measurement outliers and distance estimation errors is introduced. Meanwhile, a drift-free visual simultaneous localization and mapping (SLAM) method incorporating UWB localization for enhanced mobile robot navigation and localization performance is presented in [23]. In [24], a UWB localization system for mobile robots is proposed. Although UWB can improve the indoor localization accuracy, complex navigation environments still pose challenges to this high-precision technology.

Various filtering algorithms have been developed to fuse sensor data [25,26,27], with the Kalman filter (KF) being a representative method [28,29]. In [30], a robust error state Sage–Husa adaptive KF is proposed to enhance the UWB localization accuracy. A KF-based one-shot sim-to-real transfer learning method is investigated in [31]. The extended Kalman filter (EKF) was devised to address state estimation challenges in nonlinear dynamical systems. In [32], a decentralized EKF framework for applications based on acoustic SLAM is introduced to perform concurrent localization and environmental mapping. In [33], an EKF is proposed for estimating the position of a self-driving mobile robot to clean solar panels. However, this EKF assumes that the noise follows a Gaussian distribution, possibly being unsuitable for real-time testing in practice. To further enhance the localization accuracy, Student’s *t*-distribution can be used in the EKF [34,35]. For example, in [36], we introduce an adaptive decentralized EKF based on Student’s *t*-distribution statistics for enhanced UWB-based localization. Nevertheless, the influence of colored measurement noise (CMN) generated by the vibration of the target carrier during maneuvering has been neglected. Noted that when the target carrier runs following the planned path, specifically the robotic dog, its torso cannot be as smooth as a wheeled robot and is in a bumpy state. This bumpy state can cause interference between adjacent moments of noise, which is the effect of CMN.

To obtain stable localization information for a robotic dog, we introduce a Student’s *t*-distributed EKF for UWB localization with a switch factor under CMN, as presented in the next section. First, the design of the distributed UWB localization framework is presented. Then, the Student’s *t*-distributed EKF under CMN (cEKF) with a switch factor is proposed. Finally, practical implementation testing validates the performance of the developed method. This comprehensive research and its systematic experimental results can notably contribute to robotics, control engineering, and autonomous navigation.

- A distributed UWB localization framework is introduced. It employs the position and velocity of a robotic dog as the state vector and the range measurement from a UWB reference node (RN) to the target blind node (BN) as the measurement vector. Filter submodules independently determine the position information of the target robotic dog, while the main filter performs hierarchical data fusion of the distributed estimates.
- The Student’s *t*-distributed cEKF using a switch CMN factor is derived. Specifically, we derive the EKF under CMN considering the Student’s *t*-distribution. Then, we employ CMN factor settings and design a switch scheme for the Student’s *t*-distributed cEKF.
- We employ three kind of paths, with one path being repeatedly tested across four trials, to evaluate the developed localization method.

The remainder of this paper is structured as follows: Section 2 examines the theoretical foundations of UWB-based localization methods for quadruped robots. Section 3 presents the design of the Student’s *t*-distributed cEKF using a switch CMN factor. The experimental evaluations and their results are presented in Section 4. Finally, our conclusions are drawn in Section 5.

## 2. UWB Localization for Robotic Dog

This section outlines the technical framework and system architecture for implementing a UWB localization method for robotic dogs. In addition, we detail the state and measurement equations employed in the proposed filtering approach.

### 2.1. Indoor Integrated Localization

**Figure 1:**
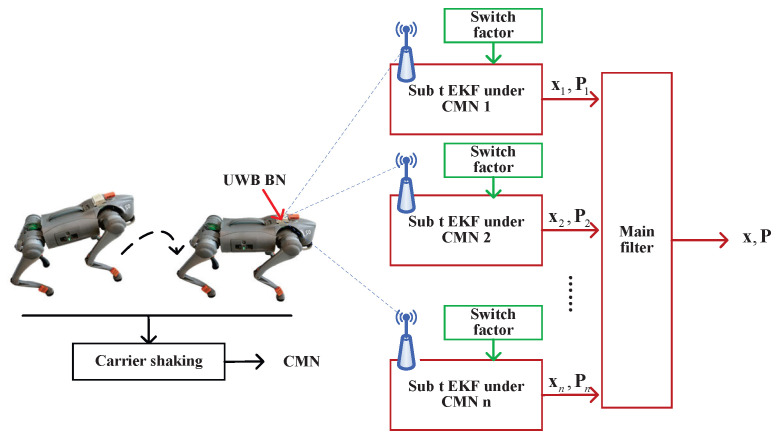
Diagram of UWB localization method for robotic dog using Student’s *t*-distributed EKF with switch factor under colored measurement noise.

Figure 1 illustrates the UWB localization method for a robotic dog using the Student’s *t*-distributed EKF with switch factor under CMN. The range measurements between a BN and RNs $d_{i},i\in \left[1,n\right]$ are employed by a submodule of Student’s *t*-distributed EKF under CMN. The switch CMN factor, which is introduced in the following section, provides an accurate noise estimate for the filter submodules. The filter submodule returns $x_{i},P_{i},i\in \left[1,n\right]$. The main filter fuses the filter submodule outputs to provide aggregate output x,P.

### 2.2. State and Measurement Equations

Employing the localization framework presented in Section 2.1, we derive the state and measurement equations of the proposed Student’s *t*-distributed EKF with switch factor under CMN. The state equation of the $i^{th}$ filter submodule is given by Equation (1).

(1) $$\underset{x_{t}^{i}}{\underset{︸}{\left[\begin{array}{c} x_{E,t}^{i}\\ x_{VE,t}^{i}\\ x_{N,t}^{i}\\ x_{VN,t}^{i} \end{array}\right]}}=\underset{F}{\underset{︸}{\left[\begin{array}{cccc} 1 & \delta t & 0 & 0\\ 0 & 1 & 0 & 0\\ 0 & 0 & 1 & \delta t\\ 0 & 0 & 0 & 1 \end{array}\right]}}\underset{x_{t-1}^{i}}{\underset{︸}{\left[\begin{array}{c} x_{E,t-1}^{i}\\ x_{VE,t-1}^{i}\\ x_{N,t-1}^{i}\\ x_{VN,t-1}^{i} \end{array}\right]}}+w_{t}^{i}\;,$$

where $\left(x_{E,t}^{i},x_{N,t}^{i}\right)$ represents the robotic dog east–north coordinates at time *t*, $\left(x_{VE,t}^{i},x_{VN,t}^{i}\right)$ represents the corresponding velocity components at time *t*, δt is the sampling time, and $w_{t}^{i}$ represents the zero-mean Gaussian noise of the system characterized by covariance matrix $Q_{t}^{i}$.

As we adopt a distributed structure, the ranging measurement between a BN and the $i^{th}$ RN, $d_{t}^{i}$, is used as the $i^{th}$ measurement of the filter submodule. The measurement equation for the $i^{th}$ filter submodule is formulated as follows:

(2) $$d_{t}^{i}=\underset{h\left(x_{t}^{i}\right)}{\underset{︸}{\sqrt{{\left(x_{E,t}-x_{E,t}^{i}\right)}^{2}+{\left(x_{N,t}-x_{N,t}^{i}\right)}^{2}}}}+V_{t}^{i}\;,$$

(3) $$V_{t}^{i}=\eta V_{t}^{i}+v_{t}^{i}\;,$$

where $\left(x_{E,t}^{i},x_{N,t}^{i}\right)$, $V_{t}^{i}$ represents the colored *Gauss–Markov* noise, η represents the CMN factor, and $v_{t}^{i}∼N\left(0,R_{t}^{i}\right)$ represents the measurement noise.

To mitigate the impact of CMN, we establish the following measurement equation:

(4) $$\begin{array}{ccc} \rho_{t}^{i} & = & \underset{\bar{h}\left(x_{t}^{i}\right)}{\underset{︸}{h\left(x_{t}^{i}\right)-\eta h\left(x_{t-1}^{i}\right)}}+V_{t}^{i}-\eta V_{t-1}^{i}\\ & = & H_{t}^{i}x_{t}^{i}+V_{t}^{i}-\eta H_{t-1}^{i}x_{t-1}^{i}-\eta V_{t-1}^{i}. \end{array}$$

Using $x_{t-1}^{i}$ and $V_{t-1}^{i}$ from Equation (1), we obtain the following:

(5) $$\rho_{t}^{i}=G_{t}^{i}x_{t}^{i}+V_{t}^{i}\;,$$

where $H_{t}^{i}=\frac{\partial h\left(x_{t}^{i}\right)}{\partial x_{t}^{i}}$ and

(6) $$G_{t}^{i}=H_{t}^{i}-\Gamma_{t}^{i}\;,$$

(7) $$\Gamma_{t}^{i}=\eta H_{t-1}^{i}F^{-1}\;,$$

(8) $$V_{t}^{i}=\Gamma_{t}^{i}w_{t}^{i}+v_{t}^{i}\;.$$

The noise component has the following properties:   

(9) $${\bar{R}}_{t}^{i}=E\left[V_{t}^{i}{\left(V_{t}^{i}\right)}^{T}\right]=\Gamma_{t}^{i}\underset{\Theta_{t}^{i}}{\underset{︸}{Q_{t}^{i}{\left(\Gamma_{t}^{i}\right)}^{T}}}+R_{t}^{i}\;\;.$$

The model is built based on Equations (1)–(4) for the $i^{th}$ filter submodule.

## 3. Student’s t-Distributed EKF with Switch CMN Factor

In this section, we present the proposed Student’s *t*-distributed EKF with switch CMN factor. First, the EKF under CMN is proposed. Second, the Student’s *t*-distributed EKF is introduced. Third, the filter under CMN is derived. Finally, the filter under CMN is modified using the switch CMN factor.

### 3.1. EKF Under CMN

We detail the EKF under CMN. In the real system modeled by Equations (1) and (2), white noise may become colored (correlated) after passing through band-limited channels [37], possibly reducing the localization accuracy of the filtering method. To mitigate this problem, we modify data fusion from the model given by Equations (1) and (2) to that given by Equations (1) and (4). Accordingly, the $i^{th}$ EKF submodule under CMN proceeds as described in Algorithm 1.
**Algorithm 1:** $i^{th}$ EKF submodule under CMN for model given by Equations (1) and (4)**Data**: $d_{t}^{i}$, ${\hat{x}}_{t}^{i}$, ${\hat{P}}_{t}^{i}$, $Q_{0}^{i}$, $R_{0}^{i}$, η**Result**: ${\hat{x}}_{t}^{i}$, ${\hat{P}}_{t}^{i}$
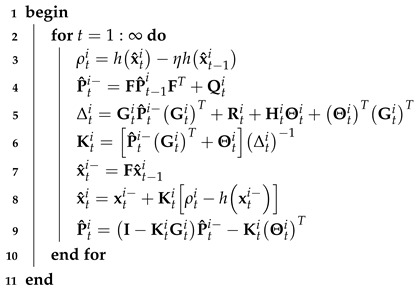


### 3.2. Student’s *t*-Distributed EKF

In [36], we proposed a Student’s *t*-distributed EKF. Here, we introduce the Student’s *t*-distributed EKF using the localization method proposed in Section 2.1. This method originally assumes white Gaussian noise. However, in practice, noise often exhibits heavy tails; thus, we assume Student’s *t*-distributed noise [38]. Based on the model given by Equations (1) and (4), we first assume the joint density to be defined by Equation (10).

(10) $$p\left(x_{t}^{i},V_{t}^{i}|\rho_{1:t}^{i}\right)=St\left(\left[\begin{array}{c} x_{t}^{i}\\ V_{t}^{i} \end{array}\right];\left[\begin{array}{c} {\hat{x}}_{t}^{i}\\ 0 \end{array}\right],\left[\begin{array}{cc} P_{t}^{i} & 0\\ 0 & Q_{t}^{i} \end{array}\right],\alpha_{t}^{i}\right)\;,$$

where $\alpha_{t}^{i}$ represents the number of degrees of freedom. We compute the one-step estimation under CMN using Equations (11) and (12).   

(11) $${\hat{x}}_{t}^{i-}={F\hat{x}}_{t-1}^{i}\;,$$

(12) $${\hat{P}}_{t}^{i-}={F\hat{P}}_{t-1}^{i}F^{T}+Q_{t}^{i}\;.$$

For measurement update, we can compute the joint probability density function of the measurement using Equation (13). Unlike the method proposed in [36], we compute the joint density under CMN.

(13) $$p\left(x_{t}^{i},e_{t}^{i}|\rho_{1:t-1}^{i}\right)=St\left(\left[\begin{array}{c} x_{t}^{i}\\ e_{t}^{i} \end{array}\right];\left[\begin{array}{c} {\hat{x}}_{t}^{i}\\ 0 \end{array}\right],\left[\begin{array}{cc} P_{t}^{i-} & 0\\ 0 & {\bar{R}}_{t}^{i} \end{array}\right],\alpha_{t-1}^{i}\right)\;,$$

where $e_{t}^{i}$ represents the innovation error. Thus, the joint statistical density is formulated as follows:

(14) $$p\left(x_{t}^{i},\rho_{t}^{i}|\rho_{1:t-1}^{i}\right)=St\left(\left[\begin{array}{c} x_{t}^{i}\\ \rho_{t}^{i} \end{array}\right];\left[\begin{array}{c} {\hat{x}}_{t}^{i}\\ h\left({\hat{x}}_{t}^{i}\right) \end{array}\right],\left[\begin{array}{cc} P_{t}^{i-} & P_{t}^{i-}{\left(G_{t}^{i}\right)}^{T}\\ G_{t}^{i}P_{t}^{i-} & \Delta_{t}^{i} \end{array}\right],\alpha_{t-1}^{i}\right)\;,$$

where the filter parameters can be computed using Equations (15)–(19).

(15) $$\alpha_{t}^{i}=\alpha_{t-1}^{i}+D_{\rho_{t}^{i}}\;,$$

(16) $${\hat{x}}_{t}^{i}={\hat{x}}_{t}^{i-}+P_{t}^{i-}{\left(G_{t}^{i}\right)}^{T}{\left(\Delta_{t}^{i}\right)}^{-1}\left(\rho_{t}^{i}-h\left({\hat{x}}_{t}^{i-}\right)\right)\;,$$

(17) $$P_{t}^{i}=\frac{\alpha_{t-1}^{i}+\delta_{\rho_{t}^{i}}^{2}}{\alpha_{t-1}^{i}+D_{\rho_{t}^{i}}}\left(P_{t}^{i-}-P_{t}^{i-}{\left(G_{t}^{i}\right)}^{T}{\left(\Delta_{t}^{i}\right)}^{-1}G_{t}^{i}P_{t}^{i-}\right)\;,$$

(18) $$\delta_{\rho_{t}^{i}}^{2}={\left(\rho_{t}^{i}-h\left({\hat{x}}_{t}^{i-}\right)\right)}^{T}{\left(\Delta_{t}^{i}\right)}^{-1}\left(\rho_{t}^{i}-h\left({\hat{x}}_{t}^{i-}\right)\right)\;.$$

Based on the model given by Equations (1) and (4), the $i^{th}$ Student’s *t*-distributed EKF submodule under CMN proceeds as described in Algorithm 2.    
**Algorithm 2:** $i^{th}$ Student’s *t*-distributed EKF submodule under CMN for model given by Equations (1) and (4)**Data**: $d_{t}^{i}$, ${\hat{x}}_{t}^{i}$, ${\hat{P}}_{t}^{i}$, $Q_{0}^{i}$, $R_{0}^{i}$, η**Result**: ${\hat{x}}_{t}^{i}$, ${\hat{P}}_{t}^{i}$
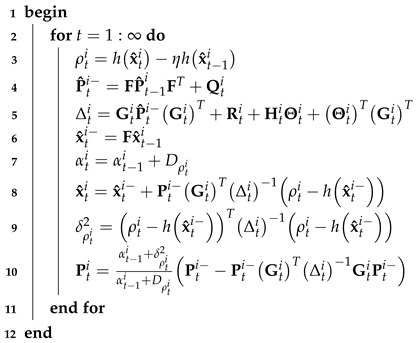


### 3.3. Switch CMN Factor

The proposed method can reduce the impact of CMN on the filtering accuracy based on Student’s *t*-distribution. However, CMN factor $\eta_{t}^{i}$ in the measurement is a fixed value, being unsuitable for complex and changing navigation environments. To address this problem, we propose a switch CMN factor, which is introduced here.

**Figure 2:**
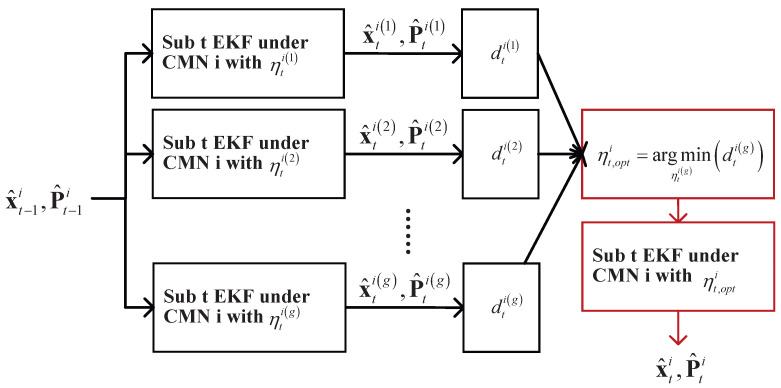
Diagram of $i^{th}$ filter submodule with noise under switch CMN factor.

A diagram of the $i^{th}$ filter submodule considering the switch noise scheme is shown in Figure 2. For the $i^{th}$ filter submodule, we employ parameter *g* for the CMN factor, which is denoted as $\eta_{t}^{i(j)},j\in \left[1,g\right]$. When the filter submodule operates, the Student’s *t*-distributed EKF operates with $\eta_{t}^{i(j)}$ and provides ${\hat{x}}_{t}^{i(j)},{\hat{P}}_{t}^{i(j)}$. We employ the *Mahalanobis* distance, which can be computed as follows:

(19) $$d_{t}^{i\left(j\right)}={\left(\rho_{t}^{i}-\bar{h}\left({\hat{x}}_{t}^{i\left(j\right)-}\right)\right)}^{T}{\bar{R}}_{t}^{i}\left(\rho_{t}^{i}-\bar{h}\left({\hat{x}}_{t}^{i\left(j\right)-}\right)\right),j\in \left[1,g\right]\;.$$

Using $d_{t}^{i\left(j\right)}$, the optimal CMN factor setting can be calculated as follows:

(20) $$\eta_{t,opt}^{i}=\underset{\eta_{t}^{i\left(g\right)}}{\arg\min}\left(d_{t}^{i\left(g\right)}\right)\;.$$

Using the optimal value of $R_{t}^{i}$, the final estimation for the filter submodule can be derived. With the local filter output, the main filter output is given by the following equations:

(21) $${\hat{P}}_{t}={\left(P_{t}^{1}\right)}^{-1}+{\left(P_{t}^{2}\right)}^{-1}+\ldots +{\left(P_{t}^{n}\right)}^{-1}\;,$$

(22) $${\hat{x}}_{t}={\hat{P}}_{t}\left[\left({\left(P_{t}^{1}\right)}^{-1}{\hat{x}}_{t}^{1}+{\left(P_{t}^{2}\right)}^{-1}{\hat{x}}_{t}^{2}+\ldots +{\left(P_{t}^{n}\right)}^{-1}{\hat{x}}_{t}^{n}\right)\right]\;.$$

Thus, the proposed method proceeds as described in Algorithm 3.    
**Algorithm 3:** Switch Student’s *t*-distributed extended Kalman filter for model given by Equations (1) and (4)**Data**: $d_{t}^{i}$, ${\hat{x}}_{t}^{i}$, ${\hat{P}}_{t}^{i}$, $Q_{0}^{i}$, $R_{0}^{i}$, $\eta_{t}^{i},i\in \left[1,g\right]$**Result**: ${\hat{x}}_{t}^{i}$, ${\hat{P}}_{t}^{i}$
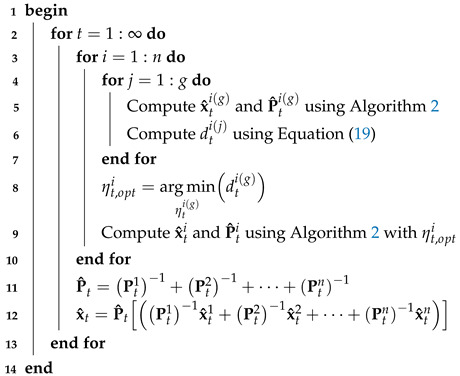


## 4. Experimental Evaluations

This section presents experimental evaluations of the proposed localization method in practical testing scenarios. Initially, the experimental configuration and testing framework are established. Subsequently, a comprehensive performance analysis of the proposed method is presented.

### 4.1. Indoor Performance Evaluation

**Figure 3:**
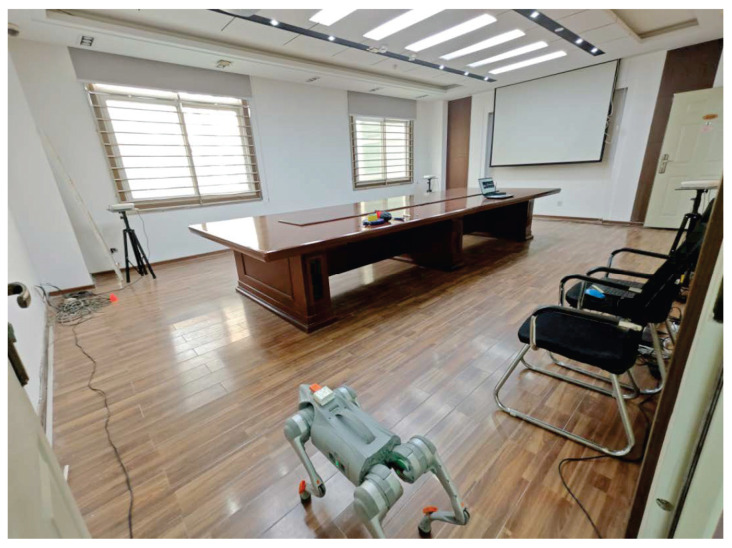
Test environment.

**Figure 4:**
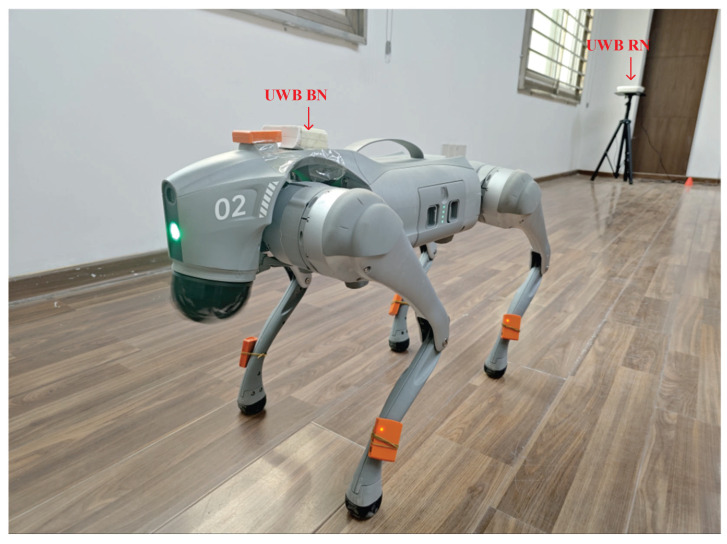
Robotic dog used in indoor test.

**Figure 5:**
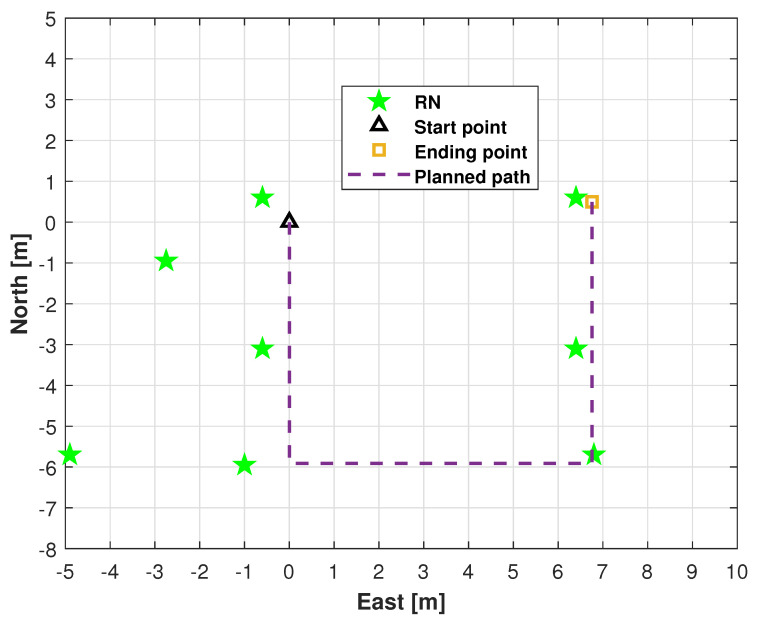
Locations of UWB RNs for testing and planned indoor test.

We conducted field tests in a high-level talent incubator overseas in Huaiyin District, Jinan City, China, as shown in Figure 3. Figure 4 shows the quadruped robot (robotic dog) employed in this study. In this work, robot dog GO2 from Yushu Technology was used as the platform and carrier of sensors. The experimental setup comprised eight RNs and a single mobile BN for position estimation. As shown in Figure 4, the UWB BN was fixed to the robotic dog, and eight UWB RNs were deployed at predetermined locations. Figure 5 shows the distribution of UWB RNs for testing.

We evaluated the performance of the proposed method experimentally. The robotic dog navigated along indoor test shown in Figure 5. Four filtering algorithms were compared with our proposal for performance benchmarking: distributed EKF, distributed UKF, distributed Student’s *t*-distributed EKF, and cEKF. For the filters, we set ${\hat{x}}_{0}^{i}={\left[\begin{array}{cccc} 0 & 0 & 0 & 0 \end{array}\right]}^{T},i\in \left[1,g\right]$, g=8, ${\hat{P}}_{0}^{i}=I_{4\times 4}$, $\eta_{t}^{1}=0.15$, $\eta_{t}^{2}=0.25$, and $\eta_{t}^{3}=0.55$. For the *t*-distributed cEKF, we set $\eta_{t}=0.3$, $R_{0}^{i}=0.002$, δt=1/20s, and the following:

(23) $$Q_{0}^{i}=\left[\begin{array}{cccc} \frac{\delta t^{2}}{4} & \frac{\delta t}{2} & 0 & 0\\ \frac{\delta t}{2} & 1 & 0 & 0\\ 0 & 0 & \frac{\delta t^{2}}{4} & \frac{\delta t}{2}\\ 0 & 0 & \frac{\delta t}{2} & 1 \end{array}\right]\times {\left(0.55\times 0.2\right)}^{2}\;.$$

**Figure 6:**
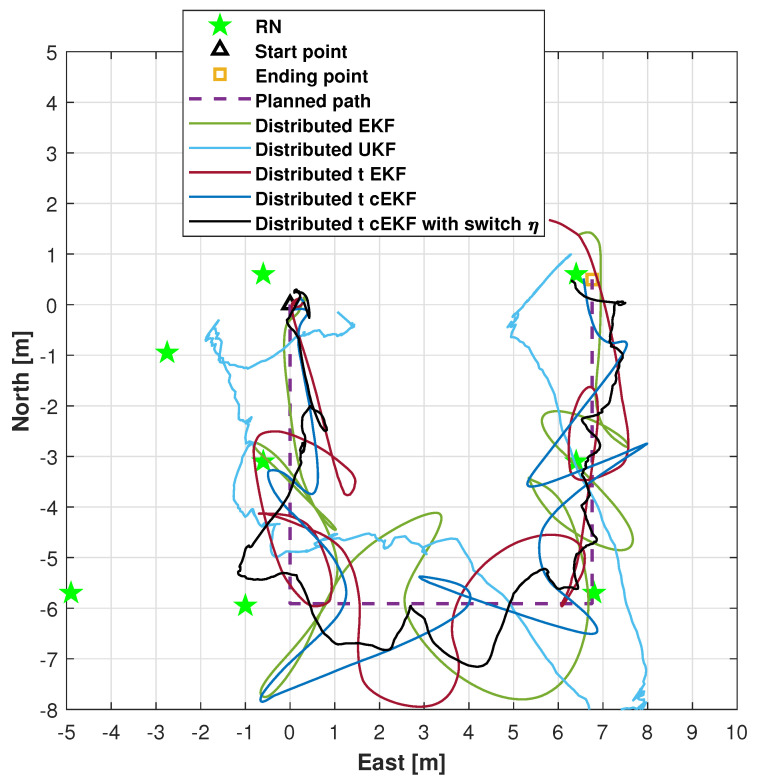
Measured trajectories along indoor test provided by distributed EKF, distributed UKF, distributed EKF with Student’s *t*-distribution, *t*-distributed cEKF, and proposed *t*-distributed cEKF with switch η during planned indoor test.

The trajectories for the indoor test obtained using the distributed EKF [39], distributed UKF [40], distributed EKF with Student’s *t*-distribution [36], *t*-distributed cEKF, and proposed *t*-distributed cEKF with switch η are shown in Figure 6. The three subfigures for the trials are included. From the figure, we can see that the proposed method can provide the closest path to the planned path, and the other methods’ solutions have obvious errors. The trajectories provided by the distributed EKF, distributed UKF, distributed EKF with *t*-distribution, and *t*-distributed cEKF show clear divergence, thus being inadequate for accurate trajectory estimation. Although the path measured by the distributed EKF with Student’s *t*-distribution diverges, its estimated trajectory demonstrates superior accuracy compared with that estimated by the distributed EKF, indicating that considering Student’s *t*-distribution is more adequate for real tests. The proposed method provides trajectory estimates with higher accuracy than the comparison methods. The proposed method can provide accurate paths in real testing. Overall, the experimental results demonstrate the superior performance of the proposed method.

**Figure 7:**
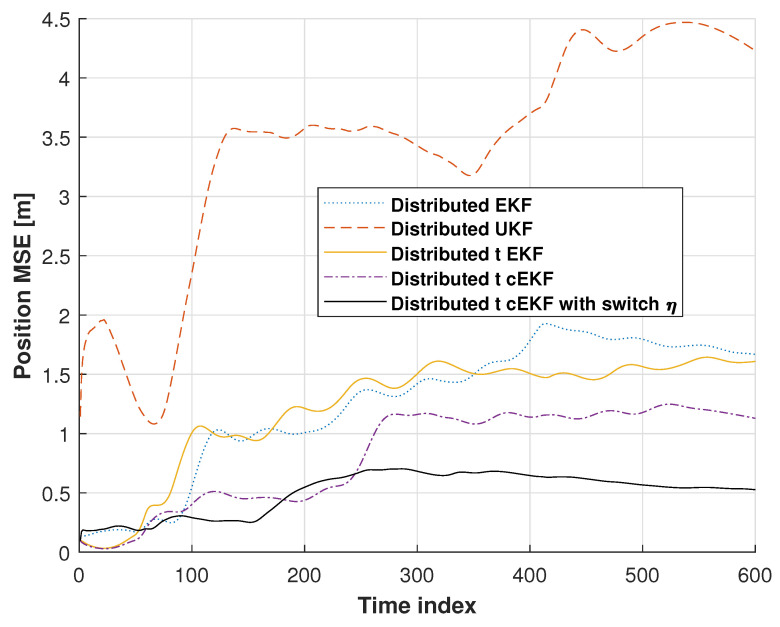
Position RMSEs from distributed EKF, distributed UKF, distributed EKF with Student’s *t*-distribution, *t*-distributed cEKF, and proposed *t*-distributed cEKF with switch η for indoor test during planned indoor test.

Figure 7 shows the position root mean square errors (RMSEs) for the distributed EKF, distributed UKF, distributed EKF with Student’s *t*-distribution, *t*-distributed cEKF, and proposed *t*-distributed cEKF with switch η. In these tests, the distributed UKF’s solution shows divergence, which has the biggest error. We can see that the distributed EKF’s RMSE is better than the distributed UKF’s RMSE, and the distributed EKF with Student’s *t*-distribution’s RMSE is better than the distributed EKF. When compared with these methods, the distributed EKF with Student’s *t*-distribution and the proposed *t*-distributed cEKF with switch η show stable RMSEs at the end of the test. The proposed method provides a smaller RMSE than the distributed EKF with Student’s *t*-distribution. From this section, we can see that our proposed method has stable performance, and it becomes the smallest error in the indoor tests.

**Figure 8:**
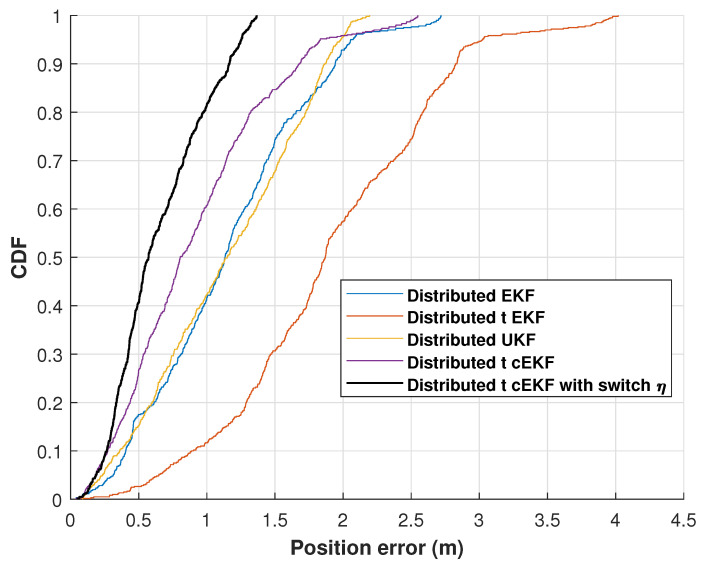
CDFs of position error obtained from distributed EKF, distributed UKF, distributed EKF with Student’s *t*-distribution, *t*-distributed cEKF, and proposed *t*-distributed cEKF with switch η for indoor test.

Figure 8 shows the cumulative distribution functions (CDFs) of the position error for the evaluated methods along the indoor test. The proposed method consistently achieves the lowest error at the 0.9 probability level throughout the four trials. The experimental findings validate the high performance of the developed method against the three comparison approaches. The position RMSEs of the distributed EKF, distributed EKF with Student’s *t*-distribution, *t*-distributed cEKF, and proposed *t*-distributed cEKF with switch η for the indoor test are listed in Table 1. It shows a substantial improvement in the mean localization accuracy, with the error decreasing from 1.43 m for the distributed UKF to 0.52 m for our method, achieving approximately 63.64% error reduction.

### 4.2. Outdoor Performance Evaluation

**Figure 9:**
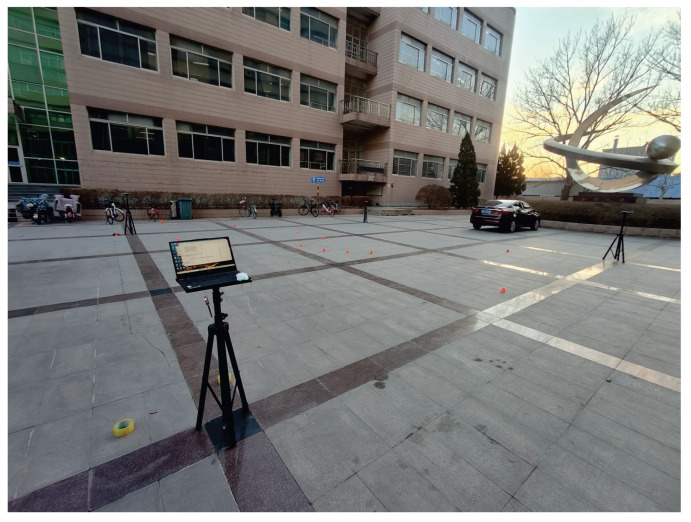
Outdoor test environment.

**Figure 10:**
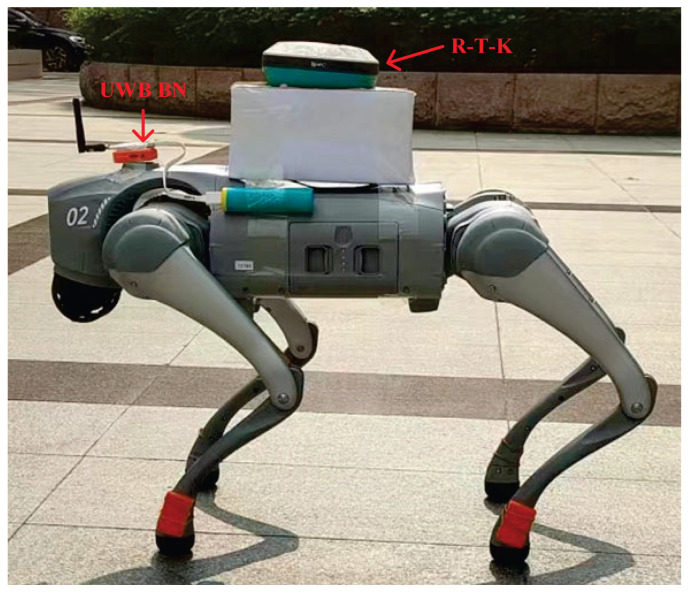
Robotic dog used in outdoor test.

**Figure 11:**
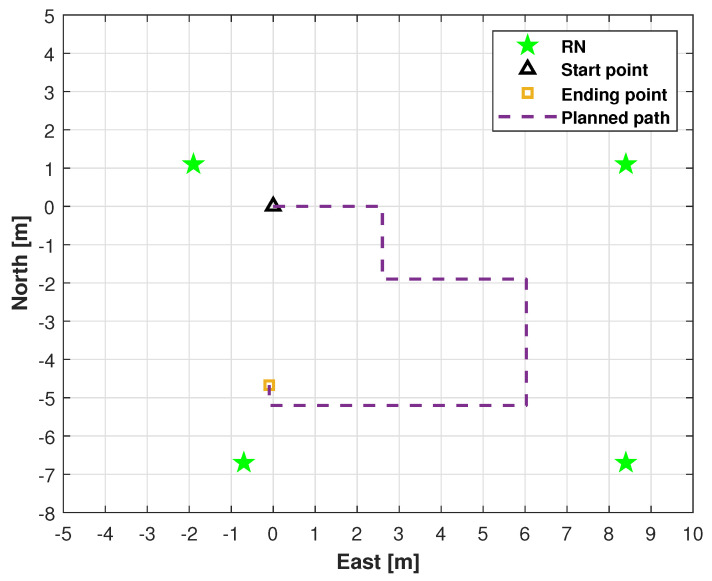
Locations of UWB RNs for testing and planned path used in outdoor test.

In this subsection, we will evaluate the performance of the proposed method in the outdoor environment. The test environment and the robotic dog used in the outdoor test are shown in Figure 9 and Figure 10. The robotic dog navigated along the path shown in Figure 11. For the filters, we set ${\hat{x}}_{0}^{i}={\left[\begin{array}{cccc} 0 & 0 & 0 & 0 \end{array}\right]}^{T},i\in \left[1,g\right]$, g=8, ${\hat{P}}_{0}^{i}=I_{4\times 4}$, $\eta_{t}^{1}=0.05$, $\eta_{t}^{2}=0.15$, and $\eta_{t}^{3}=0.3$. For the *t*-distributed cEKF, we set $\eta_{t}=0.3$, $R_{0}^{i}=0.002$, δt=1/20s, and the following:

(24) $$Q_{0}^{i}=\left[\begin{array}{cccc} \frac{\delta t^{2}}{4} & \frac{\delta t}{2} & 0 & 0\\ \frac{\delta t}{2} & 1 & 0 & 0\\ 0 & 0 & \frac{\delta t^{2}}{4} & \frac{\delta t}{2}\\ 0 & 0 & \frac{\delta t}{2} & 1 \end{array}\right]\times {\left(0.25\times 0.2\right)}^{2}\;.$$

**Figure 12:**
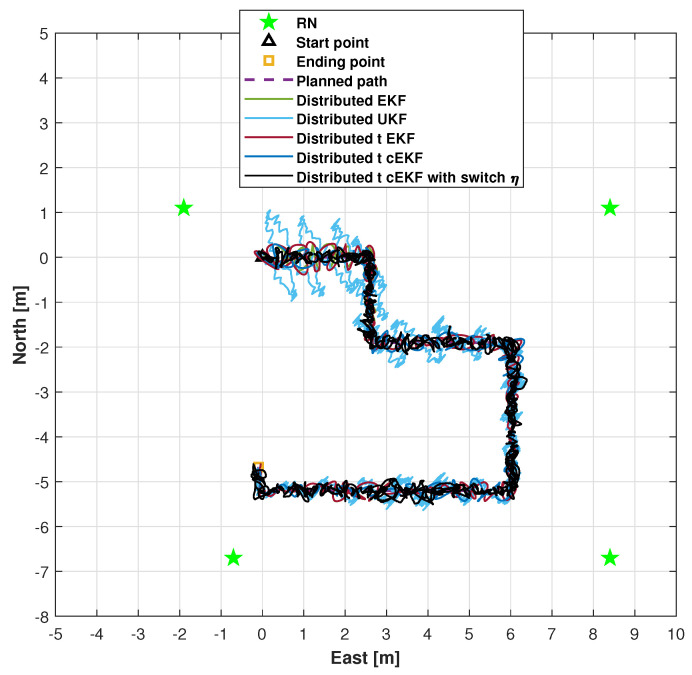
Measured trajectories along path used in outdoor test by distributed EKF, distributed UKF, distributed EKF with Student’s *t*-distribution, *t*-distributed cEKF, and proposed *t*-distributed cEKF with switch η.

**Figure 13:**
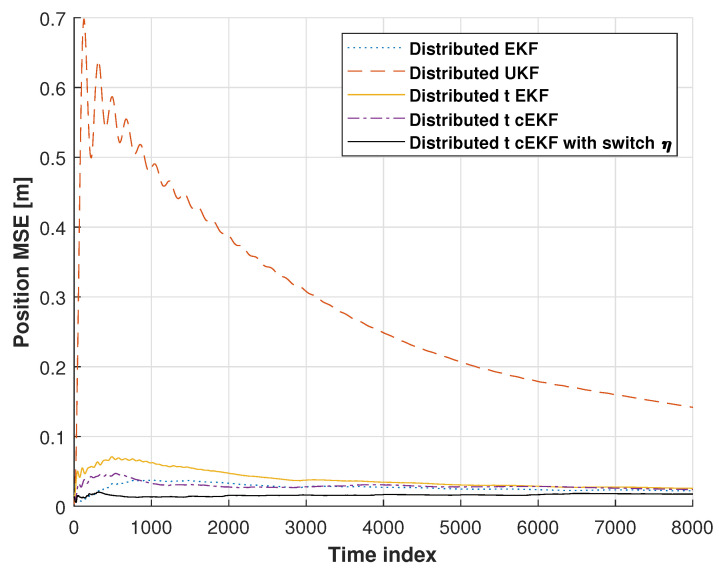
Position RMSEs from distributed EKF, distributed UKF, distributed EKF with Student’s *t*-distribution, *t*-distributed cEKF, and proposed *t*-distributed cEKF with switch η for path used in outdoor test.

**Figure 14:**
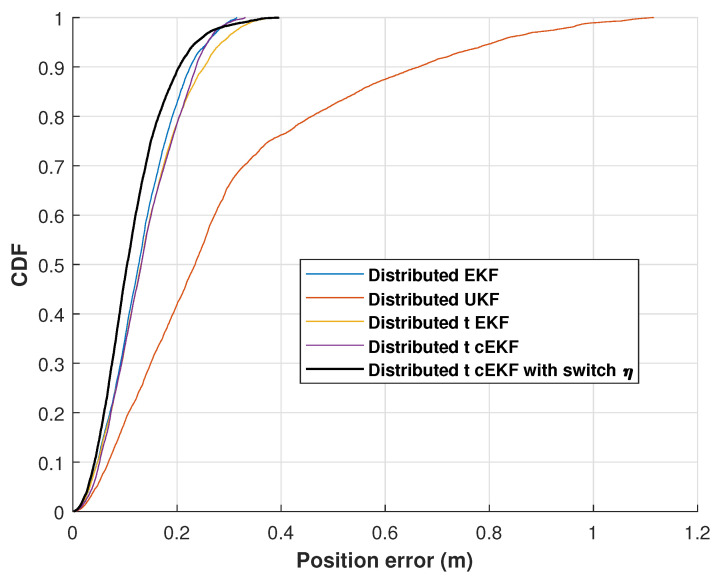
CDFs of position error obtained from distributed EKF, distributed EKF with Student’s *t*-distribution, *t*-distributed cEKF, and proposed *t*-distributed cEKF with switch η for indoor test.

Measured trajectories along the path used in the outdoor test by distributed EKF, distributed UKF, distributed EKF with Student’s *t*-distribution, *t*-distributed cEKF, and the proposed *t*-distributed cEKF with switch η are shown in Figure 12. From the figure, we can see that the trajectory measured by the distributed UKF has some big errors. Meanwhile, the other filters’ trajectories are similar. The position RMSEs from distributed EKF, distributed UKF, distributed EKF with Student’s *t*-distribution, *t*-distributed cEKF, and proposed *t*-distributed cEKF with switch η for the path used in the outdoor test are shown in Figure 13. We can see that the proposed method has the smallest RMSEs when compared with the other filters. Figure 14 shows the CDFs of position error obtained from distributed EKF, distributed EKF with Student’s *t*-distribution, *t*-distributed cEKF, and the proposed *t*-distributed cEKF with switch η for the path used in the outdoor test, and the proposed method has the smallest error at 0.9 point. Table 2 lists the position RMSEs of distributed EKF, distributed UKF, distributed EKF with Student’s *t*-distribution, *t*-distributed cEKF, and proposed *t*-distributed cEKF with switch η for outdoor test. It can be seen that the proposed method has the smallest error. Overall, the comprehensive evaluations demonstrate the exceptional performance of our proposal under practical testing conditions.

### 4.3. The Performance with Different η

**Figure 15:**
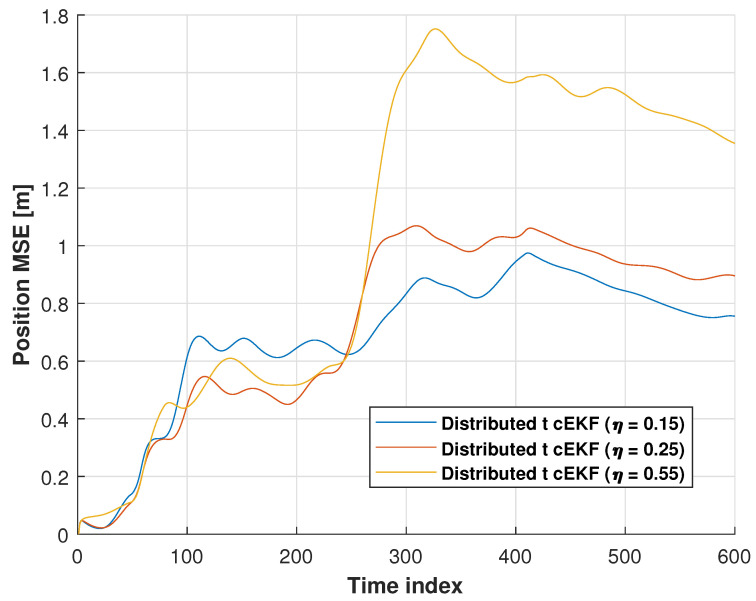
CDFs of position error obtained from distributed EKF, distributed UKF, distributed EKF with Student’s *t*-distribution, *t*-distributed cEKF, and proposed *t*-distributed cEKF with switch η for path used in indoor test.

In this subsection, we will investigate the performance with different η, which is shown in Figure 15. Table 3 lists the position RMSEs of *t*-distributed cEKF with different η for first trials in the indoor test. From the table, we can see that the *t*-distributed cEKF with different η shows different performances.

### 4.4. Running Time

In this subsection, we will investigate the running time of the methods mentioned above. In this work, we employed Matlab 2021b to run the code, and the computer we used in this work was the Lenove X1 Carbon; its RAM is 32 GB, CPU is Intel(R) Core (TM) Ultra 7 155 H, and operates with 1.4 GHz. The running times of distributed EKF, distributed UKF, distributed EKF with Student’s *t*-distribution, *t*-distributed cEKF, and proposed *t*-distributed cEKF with switch η are listed in Table 4. Note that the sampling time is 0.05 s in this work, and we can see that all the methods’ running times are lower than the sampling time.

### 4.5. Observability

The observability of the data fusion model used in this work will be discussed in this subsection. For the model (1) and (2), the observability can be computed by using the following equation [26,41]:

(25) $$rank\left({\bar{O}}_{t}\right)=rank\left(\left[\begin{array}{c} H_{t}\\ H_{t}F\\ H_{t}F^{2}\\ H_{t}F^{2} \end{array}\right]\right)\;.$$

where $H_{t}=\frac{\partial h\left(x_{t}^{i}\right)}{\partial x_{t}^{i}}=\left[\begin{array}{cccc} H_{11} & 0 & H_{13} & 0 \end{array}\right]$. Thus, if the UWB RNs are not in a straight line, $rank\left({\bar{O}}_{t}\right)=4$, the system is observable.

## 5. Conclusions

To achieve stable localization for a robotic dog, we propose a *t*-distributed cEKF with a switch factor for UWB localization. The distributed UWB localization framework is designed, and the *t*-distributed EKF under CMN with switch CMN factor is proposed. Experimental validation conducted in two indoor and outdoor practical test scenarios demonstrates the performance of the proposed method. In fact, the experimental results demonstrate the proposed algorithm’s capability to drastically reduce localization errors. The comprehensive experimental results, quantitative performance metrics, and statistical analysis outcomes confirm the effectiveness, robust performance, and practical applicability of the proposed method across multiple evaluation criteria and testing scenarios.

## Figures and Tables

**Table 1:** Position RMSEs of distributed EKF, distributed UKF, distributed EKF with Student’s *t*-distribution, *t*-distributed cEKF, and proposed *t*-distributed cEKF with switch η for indoor test.

Method	RMSE (m)		
	**East Direction**	**North Direction**	**Mean**
Distributed EKF	0.66	1.10	0.88
Distributed UKF	1.21	1.66	1.43
Distributed EKF with Student’s *t*-distribution	0.55	1.14	0.85
*t*-distributed cEKF	0.78	0.72	0.75
*t*-distributed cEKF with switch η	0.54	0.49	0.52

**Table 2:** Position RMSEs of distributed EKF, distributed UKF, distributed EKF with Student’s *t*-distribution, *t*-distributed cEKF, and proposed *t*-distributed cEKF with switch η for the outdoor test.

Method	RMSE (m)		
	**East Direction**	**North Direction**	**Mean**
Distributed EKF	0.08	0.11	0.10
Distributed UKF	0.19	0.32	0.26
Distributed EKF with Student’s *t*-distribution	0.09	0.12	0.11
*t*-distributed cEKF	0.09	0.12	0.11
*t*-distributed cEKF with switch η	0.08	0.09	0.09

**Table 3:** Position RMSEs of *t*-distributed cEKF with different η for first trial in indoor test.

η	RMSE (m)		
	**East Direction**	**North Direction**	**Mean**
0.15	0.61	0.62	0.62
0.25	0.67	0.66	0.67
0.55	0.80	0.85	0.83

**Table 4:** Running time of distributed EKF, distributed UKF, distributed EKF with Student’s *t*-distribution, *t*-distributed cEKF, and proposed *t*-distributed cEKF with switch η.

Method	Indoor Test (μs)	Outdoor Test (μs)
Distributed EKF	74.35	50.53
Distributed UKF	398.40	325.52
Distributed EKF with Student’s *t*-distribution	61.03	48.39
*t*-distributed cEKF	83.85	60.75
*t*-distributed cEKF with switch η	139.51	108.67

## Data Availability

No additional data were generated or examined in this study. The sharing of data is not relevant to this article.

## Abbreviations

The manuscript employs the following abbreviations:

BDS	BeiDou navigation satellite system
BN	Blind node
CDF	Cumulative distribution function
CMN	Colored measurement noise
EKF	Extended Kalman filter
KF	Kalman filter
RFID	Radiofrequency identification
RMSE	Root mean square error
RN	Reference node
SLAM	Simultaneous localization and mapping
UWB	Ultrawide band
